# The crystal structure of DynF from the dynemicin-biosynthesis pathway of *Micromonospora chersina*

**DOI:** 10.1107/S2053230X21012322

**Published:** 2022-01-01

**Authors:** Abigael J. Kosgei, Mitchell D. Miller, Minakshi Bhardwaj, Weijun Xu, Jon S. Thorson, Steven G. Van Lanen, George N. Phillips

**Affiliations:** a Department of Biosciences, Rice University, Houston, TX 77251, USA; b Department of Pharmaceutical Sciences, College of Pharmacy, University of Kentucky, Lexington, KY 40506, USA; c Center for Pharmaceutical Research and Innovation, College of Pharmacy, University of Kentucky, Lexington, KY 40506, USA; d Department of Chemistry, Rice University, Houston, TX 77251, USA

**Keywords:** dynemicin, natural products, biosynthetic gene clusters, enediynes, poly­ketides, anthraquinone, β-barrel, unknown function, *Micromonospora chersina* ATCC53710

## Abstract

Dynemicin is an enediyne natural product from *Micromonospora chersina* ATCC53710. Access to the biosynthetic gene cluster of dynemicin has enabled the *in vitro* study of gene products within the cluster to decipher their roles in assembling this unique molecule. This paper reports the crystal structure of DynF, the gene product of one of the genes within the biosynthetic gene cluster of dynemicin. DynF is revealed to be a dimeric eight-stranded β-barrel structure with palmitic acid bound within a cavity. The presence of palmitic acid suggests that DynF may be involved in binding the precursor polyene heptaene, which is central to the synthesis of the ten-membered ring of the enediyne core.

## Abstract 2

The crystal structure of DynF was determined to a resolution of 1.50 Å, revealing a dimeric eight-stranded β-barrel structure with palmitic acid bound in the interior.

## 1. Introduction

**Figure 1:**
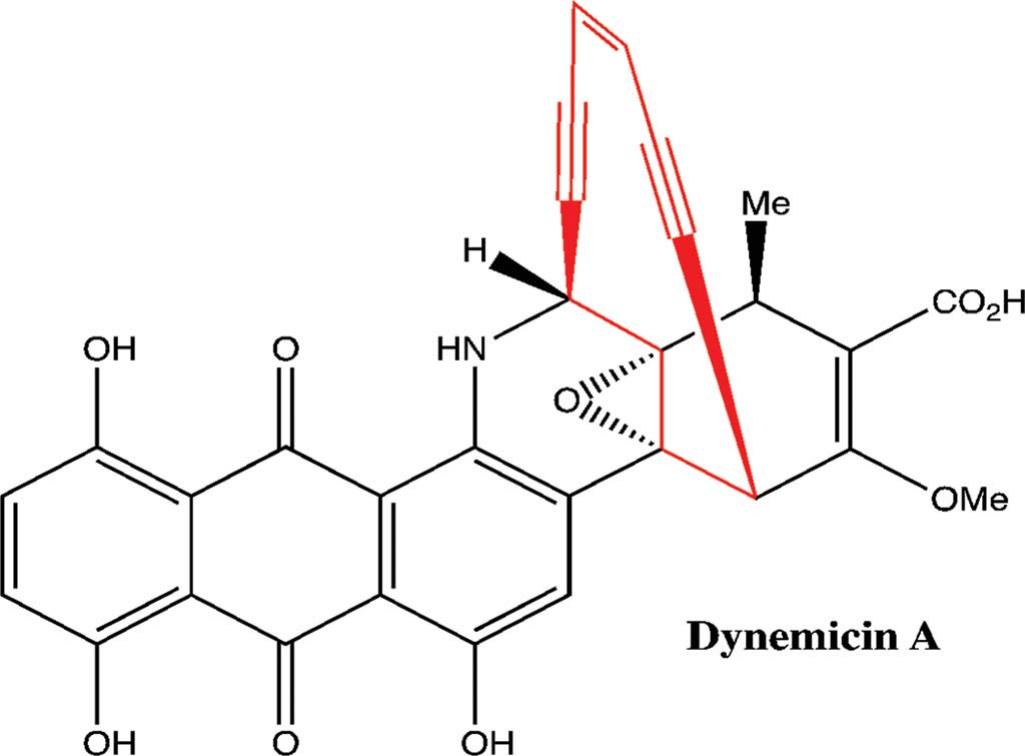
Dynemicin A has a unique molecular architecture comprising an enediyne core (red) and peripheral moieties (black). The enediyne core is the key to the cytotoxicity of dynemicin.

Dynemicin A is an anthraquinone-fused ten-membered enediyne and a metabolite of *Micromonospora chersina* ATCC53710. Like many other enediynes, dynemicin has a novel molecular architecture, a notable mode of action and potent cytotoxicity (Thorson *et al.*, 2000 ▸; Van Lanen & Shen, 2008 ▸). Dynemicin comprises an enediyne core, which is common to all enediynes, and peripheral moieties (Fig. 1 ▸), which give the enediynes structural diversity. The main ring of the dynemicin core contains two acetylenic groups conjugated to a double bond in a ten-membered carbocyle. The enediyne core structural unit (termed the warhead) is central to the fundamental mechanism of action. The anthraquinone group on the peripheral moeity of dynemicin intercalates into the minor groove of DNA to confer binding specificity (Sugiura *et al.*, 1990 ▸).

In the presence of NADPH or thiol, the quinone is reduced to hydroquinone, leading to opening of the epoxide (Avendaño & Menéndez, 2015 ▸), which triggers the activation of the enediyne core. Dynemicin then undergoes a Bergman cycloaromatization to form a reactive 1,4-dehydrobenzene bi­radical that is capable of abstracting H atoms from the DNA backbone, causing single- or double-stranded DNA scission. The high reactivity of enediynes with DNA makes them quite toxic. In natural environments, some microorganisms producing enediynes protect themselves from their cytotoxic action by a self-resistance mechanism that uses a self-sacrificing protein (Biggins *et al.*, 2003 ▸; Elshahawi *et al.*, 2014 ▸) or specialized ABC transporters to export the toxin (Beis & Rebuffat, 2019 ▸). In cancer therapy, the toxicity of enediynes has been exploited by conjugating the molecules to tumor-specific monoclonal antibodies in polymer-assisted delivery systems to prevent premature cytotoxic effects on other important cells in the body (Abe & Otsuki, 2002 ▸; Shah, 2017 ▸).

Dynemicin can be produced by organic synthesis or in *M. chersina*. The total chemical synthesis of enediynes is challenging, but has been achieved for dynemicin (Nicolaou *et al.*, 1991 ▸, 1993 ▸; Shair *et al.*, 1996 ▸; Myers *et al.*, 1995 ▸). The cloning, sequencing and identification of the biosynthetic gene clusters of dynemicin has paved the way for *in vitro* studies of these biosynthetic enzymes (Gao & Thorson, 2008 ▸). The putative biosynthesis of dynemicin is correspondingly complex: the *M. chersina* whole genome sequence analysis revealed 60 open reading frames (ORFs) within the biosynthetic gene cluster related to dynemicin production. Using homology to other characterized genomes, these genes have been annotated to suggest functions such as biosynthesis of the enediyne core, maturation and regulation, while there are also those with as yet unknown functions (Gao & Thorson, 2008 ▸). The gene cluster contains a common iterative type I polyketide synthase (PKSE) that is essential for the biosynthesis of a precursor polyene (heptaene) which, in the presence of other accessory enzymes, forms the ten-membered enediyne core of dynemicin (Liu *et al.*, 2003 ▸). Evidence has accumulated that the PKSE product is also the precursor of the anthraquinone, and the biosynthesis of this moiety proceeds through a δ-thiolactone iodoanthracene intermediate (Cohen & Townsend, 2018 ▸). While some insight into the conversion of the iodo­anthracene to the anthraquinone has recently been reported upon examination of the biosynthesis of sungeidines (Ma *et al.*, 2021 ▸), which, like dynemicin, are anthraquinone-fused ten-membered enediynes, the steps leading to the iodoanthracene in addition to the enediyne core remain mostly unknown.

This study targets one of several proteins of unknown function encoded by a gene within the biosynthetic gene cluster of dynemicin, herein named DynF. The gene encoding DynF was originally annotated *orf15* (Gao & Thorson, 2008 ▸) and was deposited as ORF18 with unknown function (GenBank accession No. ACB47058.1). The DynF ortholog Sgd15 was very recently demonstrated by gene inactivation to be essential for sungeidine biosynthesis, and no intermediates, including the iodoanthracene, were detected (Ma *et al.*, 2021 ▸), suggesting that DynF and Sgd15 are involved in the early steps of processing the PKSE product to either the enediyne core or iodoanthracene, or potentially both. Here, we report the structural characterization of DynF using X-ray crystallo­graphy as an initial step towards deciphering its role in the biosynthesis of this unique anthraquinone-fused enediyne.

## 2. Materials and methods

### 2.1. Macromolecule production

A construct for DynF from *M. chersina* ATCC53710 with a cleavable 6×His expression and purification tag on the N-terminus was codon-optimized for production in *Escherichia coli*. It was ordered as a synthetic gBlock from Integrated DNA Technologies (IDT, Coralville, Iowa, USA). Amplification of the gene was performed by polymerase chain reaction (PCR) using Q5 High-Fidelity 2× Master Mix (NEB, Westwood, Massachusetts, USA). Gibson assembly was used to clone the gene into a pNIC28-Bsa4 vector (Savitsky *et al.*, 2010 ▸; Addgene, Watertown, Masachusetts, USA). It was then transformed into DH5α competent *E. coli* cells (Invitrogen, Carlsbad, California, USA). Sanger sequencing (Genewiz, South Plainfield, New Jersey, USA) was used to verify the sequence of the construct.

The DynF_pNIC28-Bsa4 plasmid was transformed into competent *E. coli* BL21 (DE3) expression cells (Agilent Technologies, Cedar Creek, Texas, USA). 5 µl of the transformed cells were then grown overnight on Luria–Bertani (LB) agar plates containing 50 µg ml^−1^ kanamycin antibiotic. From a single colony, a seed culture of 50 ml was grown in LB medium supplemented with 50 µg ml^−1^ kanamycin to select for expression in the cells. DynF was expressed in BL21 (DE3) competent *E. coli* cells and was most soluble when induced with 0.5 m*M* isopropyl β-d-1-thiogalactopyranoside (IPTG) and cultured at 291 K after induction. To obtain suitable quantities of DynF protein for crystallization experiments, 1 l culture was grown from a 50 ml overnight seed culture until an OD_600_ of ∼0.6–0.8 was reached. The cells were then induced with 0.5 m*M* IPTG and cultured at 291 K for 17 h. These cells were harvested by centrifugation at 5000 rev min^−1^ for 40 min.

The cell pellet was resuspended using a lysis buffer consisting of 10% glycerol, 20 m*M* imidazole, 50 m*M* 4-(2-hydroxyethyl)-1-piperazineethanesulfonic acid (HEPES) pH 8.0, 500 m*M* NaCl, 1 m*M* tris(2-carboxyethyl)phosphine hydrochloride (TCEP). The cells were then lysed by sonication for 4 min 30 s pulse on and 2 min pulse off with 50% amplitude (Qsonica Q700 sonicator, Newton, Connecticut, USA). The lysed cells were centrifuged at 20 000 rev min^−1^ for 45 min. Immobilized metal-affinity chromatography (IMAC) with Ni–NTA resin (Qiagen, Hilden, Germany) was used to purify the protein. The protein was eluted from the nickel column using a buffer consisting of 10% glycerol, 500 m*M* imidazole, 25 m*M* HEPES pH 8.0, 500 m*M* NaCl, 1 m*M* TCEP. Sodium dodecyl sulfate–polyacrylamide gel electrophoresis (SDS–PAGE) was used to confirm the production of DynF protein with an expected size of 22.6 kDa. The protein was concentrated and buffer-exchanged into Tobacco etch virus (TEV) protease buffer (25 m*M* Tris pH 8.0, 150 m*M* NaCl, 1 m*M* DTT) using an Amicon ultracentrifugal filter (10 000 molecular-weight cutoff, Merck KGaA, Darmstadt, Germany). The protein concentration was obtained using a UV–Vis spectrophotometer with an extinction coefficient of 1.191, and was confirmed using a NanoDrop. The histidine tag at the N-terminus of the DynF protein was cleaved from the protein using His-tagged TEV protease. Subtractive nickel column purification was performed to remove the uncleaved protein and protease. DynF was run on a size-calibrated HiLoad 16/600 Superdex 200 pg (GE Healthcare, Uppsala, Sweden) size-exclusion column (SEC) to further purify it and analyze its oligomeric state (Supplementary Fig. S1). Macromolecule-production information is summarized in Table 1 ▸.

### 2.2. Crystallization

**Figure 2:**
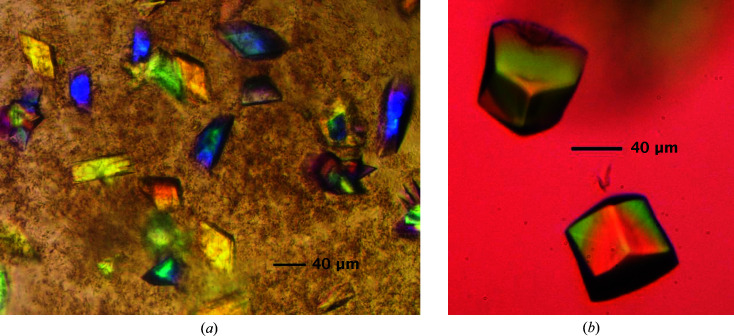
DynF crystals as viewed under an Olympus BHTP polarizing microscope equipped with an Olympus OM-D E-M5 digital camera. (*a*) Initial crystal hits observed at a 1:1 ratio of protein to precipitant. (*b*) Larger crystals were obtained by increasing the amount of protein in the crystallization wells.

DynF was concentrated to 20 mg ml^−1^ and subjected to high-throughput crystallization screening. A Mosquito LCP robot (SPT Labtech) was used with commercially available screens, including Wizard 1 and 2 and Wizard 3 and 4 (Rikagu Reagents, Bainbridge Island, Washington, USA), PEGRx and Crystal Screen HT (Hampton Research, Aliso Viejo, California, USA). Screens were set up via the sitting-drop vapor-diffusion method in MRC 2 96-well crystallization plates (Hampton Research, Aliso Viejo, California, USA) with drops consisting of 200 nl protein solution (20 mg ml^−1^) mixed with 200 nl reservoir solution. Initial crystal hits (Fig. 2 ▸ *a*) were observed in two wells of the Wizard 3 and 4 crystallization screen [C1, 10%(*w*/*v*) PEG 1000, 10%(*w*/*v*) PEG 8000; F10, 25%(*w*/*v*) PEG 1500, 0.1 *M* PCB buffer pH 7.0]. Optimization for larger crystals from well F10 of the Wizard 3 and 4 crystallization screen was achieved by increasing the protein:precipitant ratio (300:200 nl) in the initial drop (Fig. 2 ▸ *b*). Potassium iodide (KI) derivatives were prepared by soaking native DynF crystals for 30 s in 0.5 m*M* KI dissolved in the reservoir solution. Native and KI-soaked crystals were looped using Mounted CryoLoops (Hampton Research, Aliso Viejo, California, USA), cryocooled in liquid nitrogen and shipped to the Advanced Photon Source synchrotron facility at Argonne National Laboratory. Crystallization information is summarized in Table 2 ▸.

### 2.3. Data collection and processing

X-ray diffraction data were collected remotely at wavelengths of 1.0332 and 1.823 Å for the native crystals and the heavy-atom derivative crystals, respectively. The native and iodide-soaked crystals diffracted to resolutions of 1.55 and 2.38 Å, respectively. The DynF diffraction data were indexed in the orthorhombic space group *C*222_1_, with unit-cell parameters *a* = 60, *b* = 65, *c* = 131 Å. The diffraction data were integrated and scaled using *XDS* (Kabsch, 2010*a* ▸,*b* ▸). Data-collection and processing statistics are summarized in Table 3 ▸.

### 2.4. Structure solution and refinement

The SAD (single-wavelength anomalous diffraction) and SIRAS (single isomorphous replacement with anomalous scattering) methods were used to phase the structure of DynF. Scaling, substructure solution and phasing were accomplished with *SHELXC*/*D*/*E* (Sheldrick, 2010 ▸) using the *HKL*2*MAP* graphical interface (Pape & Schneider, 2004 ▸). Automated protein tracing with *ARP*/*wARP* (Langer *et al.*, 2008 ▸) was used on both the SAD and the SIRAS maps to build 418 of 422 residues.

Refinement and model building were performed using *phenix.refine* (Afonine *et al.*, 2012 ▸) and *Coot* (Emsley *et al.*, 2010 ▸). The structure was visualized using a collaborative 3D graphics system (Yennamalli *et al.*, 2014 ▸). The software applications used in this project were compiled and configured by SBGrid (Morin *et al.*, 2013 ▸). The structure of DynF and its corresponding diffraction data have been deposited in the worldwide Protein Data Bank (wwPDB; Berman *et al.*, 2003 ▸) as entry 6ubl. Refinement statistics are summarized in Table 4 ▸.

### 2.5. Ligand determination using gas chromatography–mass spectrometry (GC-MS)

Acetonitrile (0.5 ml) was added to 0.5 ml purified DynF protein (40 mg ml^−1^) and rotated at room temperature for 2 h. The mixture was centrifuged using a protein concentrator (3000 molecular-weight cutoff; Millipore, Cork, Ireland) at 4000 rev min^−1^ for 20 min, and the acetonitrile extract (100 µl) was filtered to remove any remaining insoluble debris. Gas chromatography–mass spectrometry (GC-MS) analysis was performed using an Agilent 7890 GC with a 30 m × 250 µm × 0.25 mm HP-5ms UI capillary column and an Agilent G7081B MSD. Helium was the carrier gas at a flow rate of 1 ml min^−1^. The temperatures of the injector, the interface and the liner were 523 K. Injections of 5 µl were performed in splitless mode. The column temperature was initially 343 K and was held for 2 min, increased to 513 K at 277 K min^−1^ and run for a total run time of 59.5 min. For GC–MS detection, an electron-impact ionization system was used. The mass range was set to 50–500 *m*/*z* with a filament delay of 4 min. The sample-injection volume was 1 µl.

### 2.6. Co-expression of DynF with SgcE and DynE7

The genes encoding the C-1027 polyketide synthase SgcE and dynemicin thioesterase DynE7 were cloned into pETDuet using standard procedures. The gene encoding DynF was cloned into pDB.His.MBP. All genes were sequenced to confirm their identity. Both pETDuet and pDB.His.MBP plasmids with the respective genes were co-transformed into *E. coli* BL21 (DE3) cells. The transformed cells were plated on LB agar supplemented with 50 µg ml^−1^ kanamycin and 50 µg ml^−1^ carbenicillin. After overnight incubation at 310 K, a single colony was used to inoculate 5 ml LB medium supplemented with 50 µg ml^−1^ kanamycin and 50 µg ml^−1^ carbenicillin. Incubation was performed overnight at 310 K with shaking at 250 rev min^−1^. The culture (0.5 ml) was transferred into 50 ml fresh LB supplemented with 50 µg ml^−1^ kanamycin and 50 µg ml^−1^ carbenicillin and incubated at 291 K and 250 rev min^−1^. When the optical density at 600 nm reached 0.5–0.6, expression was induced with sterile IPTG solution (final concentration 0.1 m*M*) and incubated at 291 K and 250 rev min^−1^ for an additional 36 h. The cells were centrifuged at 4000 rev min^−1^ at 277 K for 30 min, and the wet pellet was washed once with 30 ml phosphate buffer pH 7.2. The pellet was resuspended in 30 ml phosphate buffer pH 7.2 and sonicated for 2 min with 10 s pulse on and 50 s pulse off (with 40% amplitude) on ice. The lysate was purified using nickel resin and then with amylose resin to purify the MBP-tagged DynF. The sample was extracted with acetonitrile and analyzed by GC-MS as described above.

## 3. Results and discussion

**Figure 3:**
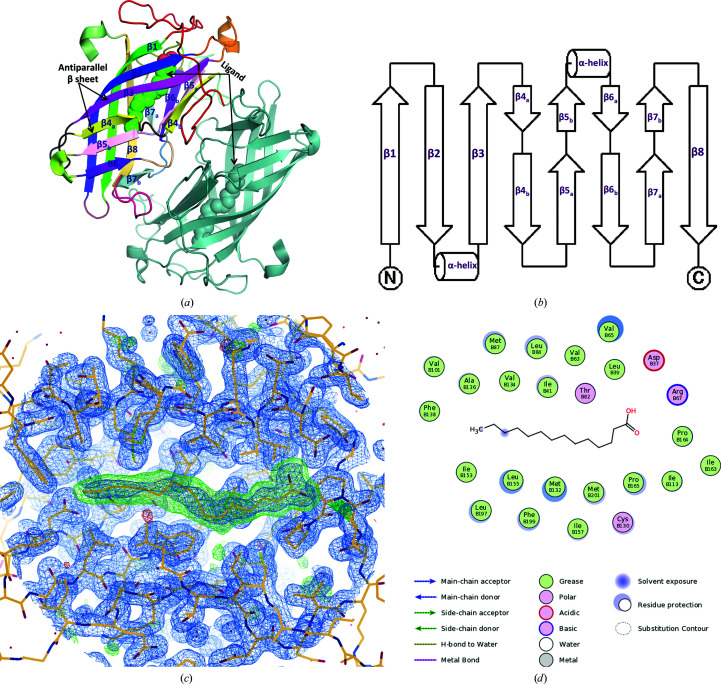
Structure of DynF. (*a*) A ribbon diagram of the crystal structure of DynF (PDB entry 6ubl) viewed down its noncrystallogaphic twofold axis. The ligand is represented as spheres embedded in the DynF β-barrels. Each protomer of DynF comprises an eight-stranded antiparallel β-barrel connected by loops and two helical segments. Strands 4, 5, 6 and 7 are interrupted by dimerization interactions. This figure was generated in *PyMOL* (version 2.4.1; Schrödinger). (*b*) A topology diagram of DynF generated using *TopDraw* (Bond, 2003 ▸) shows that each strand is connected to its next neighbor, as is typical of most eight-stranded antiparallel β-barrel structures. (*c*) The final 2*mF*_o_ − *mF*_c_ map (contoured at 1 r.m.s.d) in blue with ±3 r.m.s.d. contours of the polder omit map in green/red represented in *Coot*. The green positive difference density clearly shows the location of the bound ligand (palmitic acid) that was omitted from the model phases for the polder map calculation. (*d*) The ligand in the DynF structure is surrounded by hydrophobic amino-acid groups, except for residues that interact with the carboxylic group at one end of the palmitic acid. This figure was generated with the *FLEV* (*Flatland Ligand Environment View*) tool in *Coot*.

The structure of DynF was determined to a resolution of 1.50 Å with a final *R*_work_ and *R*_free_ of 0.16 and 0.19, respectively. The DynF crystal has two molecules in its asymmetric unit (Fig. 3 ▸ *a*). An analysis of the crystal contacts with *PISA* (Krissinel & Henrick, 2007 ▸) predicts that DynF forms a stable dimer. Size-exclusion chromatography (SEC) showed that DynF could exist in a higher oligomeric state in solution (Supplementary Fig. S1).

DynF is comprised of antiparallel β-sheets that are connected by short loops and helical segments to form eight-stranded antiparallel β-barrels (Supplementary Fig. S2). The β-strands of the DynF structure are labeled 1, 2, 3, 4a/4b, 5a/5b, 6a/6b, 7a/7b and 8. Two short helical segments connect β-strands 2/3 and 5b/6a. The topology of DynF shows that each strand is directly linked to its neighbor, which is typical of most topologies of eight-stranded antiparallel β-barrels (Fig. 3 ▸ *b*) such as outer membrane proteins, avidins, fatty-acid binding proteins and lysozyme inhibitors (Geerds *et al.*, 2014 ▸). Deviating from a canonical eight-stranded, up–down β-barrel (Lalonde *et al.*, 1994 ▸), we observed short loops inserted within strands 4, 5, 6 and 7 (Figs. 3 ▸ *a* and 3 ▸ *b*). Some of the loop residues within strands 6 and 7 are located at the dimer interface and thus may be involved in stabilization of the dimer (Supplementary Fig. S3, Supplementary Tables S1 and S2).

Comparative analysis of protein structures can help to decipher their functions. The DynF structure was compared with other structures in the Protein Data Bank (PDB) using the *DALI* server (Holm, 2020 ▸). DynF did not have significant structural similarities to previous structures (*Z*-score of <10; structural identity of <15%; Supplementary Table S3, Supplementary Fig. S4), suggesting that DynF represents a novel fold, thus lacking precedents for providing a known mechanism.

The electron-density map of DynF revealed an elongated ligand embedded within the β-barrel (Fig. 3 ▸ *c*). The size and shape of the ligand, as well as its binding environment, are suggestive of a 16-carbon fatty acid, which was confirmed by gas chromatography–mass spectrometry (GC-MS) as palmitic acid (Supplementary Fig. S5). Palmitic acid is the most abundant fatty acid expressed in *E. coli* cells (Cronan & Thomas, 2009 ▸). We hypothesize that the ligand co-purified with the protein. The surface representation of DynF in *PyMOL* reveals that the binding cavity of the ligand is closed and may thus require the channel within the ligand-binding site to open and close in order to allow the ligand to enter and exit.

The hydrophobicity of the side chains of the residues in the ligand-binding cavity (Fig. 3 ▸ *d*) and the presence of palmitic acid suggest that DynF may be involved in the binding or processing of the PKSE product or an early biosynthetic intermediate, which is consistent with the results from the inactivation of *sgd15* (Ma *et al.*, 2021 ▸). A linear conjugated heptaene has previously been observed upon heterologous co-expression of the genes encoding PKSE and a type II thioesterase in *E. coli* (Annaval *et al.*, 2017 ▸; Smanski *et al.*, 2011 ▸), and was hypothesized to potentially serve as the DynF ligand or substrate. To test this, DynF was co-expressed with a C-1027 polyketide synthase, SgcE, and a dynemicin thio­estarase, DynE7. However, GC-MS analysis of the ligand following purification of DynF after co-expression of the three genes gave an identical profile predominantly containing palmitic acid. Thus, the role of DynF has not directly been confirmed. Nonetheless, the structural data now provide insight into its potential role in binding the precursor polyene and provide an opportunity to further explore this role via *in vitro* assays.

## Supplementary Material

PDB reference: DynF from *Micromonospora chersina*, 6ubl

Supplementary Figures and Tables. DOI: 10.1107/S2053230X21012322/ft5115sup1.pdf

## Figures and Tables

**Table 1:** Macromolecule-production information

Source organism	*Micromonospora chersina*
DNA source	*E. coli* codon-optimized synthesis (gBlock) from Genewiz
Insert forward primer†	5′-agaacctgtacttccaatccATGTCGACTAAATCTGTATT-3′
Insert reverse primer	5′-ACCAGACCAGATGTGAacggtctccagtaaaggtgg-3′
Vector forward primer	5′-TTAAACCAGACCAGATGTGAacggtctccagtaaaggtg-3′
Vector reverse primer	5′-agaacctgtacttccaatccATGTCGACTAAATCTGTATT-3′
Expression vector‡	pNIC28-Bsa4
Expression host	*Escherichia coli* BL21 (DE3)
Complete amino-acid sequence of the construct produced§	mgsshhhhhhenlyfq/sMSTKSVLFGRPVQTEGVPNVYAGAPVVPWTPPEPGIDNLGINSIDTFAVPGVGEYTVAFDGWVRVVRSPSTSGEWADAEVYTNLIEMKMVGECEELGKITVTLNPDCLSAGQIRTPFDPYAGEGPSAKACRMAVGAIFDMPKLGLKLMNREPIILTIDDVRSIPPAGAPGKGQIYRMMPLLDVNDPDGQPVAYLTSLRFNMGGYLKPDQM

† The primers contained overhangs for cloning, which are represented as lower case letters.

‡ Plasmid pNIC28-Bsa4 was a gift from Opher Gileadi (Addgene plasmid #26103).

§ The expression and purification tag is shown in lower case with the TEV recognition site underlined and the cut site indicated with a slash.

**Table 2:** Crystallization

Method	Vapor diffusion, sitting drop
Plate type	MRC 2 (96-well crystallization plate)
Temperature (K)	293
Protein concentration (mg ml^−1^)	20
Buffer composition of protein solution (crystallization buffer)	0.025 *M* Tris pH 8.0, 0.15 *M* NaCl, 1 m*M* DTT
Composition of reservoir solution	25%(*w*/*v*) PEG 1500, 0.1 *M* PCB buffer pH 7.0
Volume and ratio of drop†	300 nl (1.5:1)
Volume of reservoir (µl)	50

† The initial screening for crystals was performed using a 1:1 ratio of protein to reservoir solution.

**Table 3:** Data collection and processing Values in parentheses are for the outer shell.

	Native	KI soaked
Diffraction source	Beamline 23-ID-B, APS	Beamline 23-ID-B, APS
Wavelength (Å)	1.0332	1.8233
Temperature (K)	100	100
Detector	Dectris EIGER X 16M	Dectris EIGER X 16M
Crystal-to-detector distance (mm)	219.1	199.4
Rotation range per image (°)	0.2	0.2
Total rotation range (°)	180	360
Exposure time per image (s)	0.2	0.2
Space group	*C*222_1_	*C*222_1_
*a*, *b*, *c* (Å)	59.83, 116.13, 129.88	59.93, 116.08, 131.03
Mosaicity (°)	0.14	0.15
Resolution range (Å)	34.7–1.50 (1.59–1.50)	49.3–2.38 (2.53–2.40)
Total No. of reflections	440004 (21559)	198086 (8709)
No. of unique reflections	70922 (5926)	17513 (1990)
Completeness (%)	97.6 (87.1)	93.7 (66.8)
Multiplicity	6.2 (3.9)	11.3 (4.4)
〈*I*/σ(*I*)〉	14.1 (0.63)†	14.1 (1.8)‡
*R*_meas_	0.061 (1.90)	0.120 (0.47)
CC_1/2_	1.00 (0.37)	0.998 (0.88)
Overall *B* factor from Wilson plot (Å^2^)	32	50

† 〈*I*/σ(*I*)〉 falls below 2.0 at 1.67 Å resolution.

‡ 〈*I*/σ(*I*)〉 falls below 2.0 at 2.47 Å resolution.

**Table 4:** Structure solution and refinement Values in parentheses are for the outer shell.

PDB entry	6ubl
Resolution range (Å)	34.7–1.50 (1.52–1.50)
Completeness (%)	97.3
No. of reflections, working set	70715 (2000)
No. of reflections, test set	3518 (117)
Final *R*_work_	0.161 (0.405)
Final *R*_free_	0.185 (0.362)
No. of non-H atoms	
Protein	3287
Ligand	3
Solvent	422
Total	374
Ligand RSC	
Palmitic acid A	0.94
Palmitic acid B	0.91
R.m.s. deviations	
Bonds (Å)	0.007
Angles (°)	1.0
Average *B* factors (Å^2^)	32
Protein	31
Ligand	46
Water	41
Ramachandran plot	
Most favored (%)	99.3
Allowed outliers (%)	0.0
