# Supplementary material for: The crystal structure of DynF from the dynemicin-biosynthesis pathway of Micromonospora chersina
Source: Acta Crystallogr F Struct Biol Commun. 2022 Jan 1;78(Pt 1):1–7. doi: 10.1107/S2053230X21012322 (PMC8725005; doi:10.1107/S2053230X21012322)
Supplement: Supplementary file 1 [file f-78-00001-sup1.pdf]

Acta Cryst F
STRUCTURAL BIOLOGY
COMMUNICATIONS

**Volume 77 (2021)**

**Supporting information for article:**

# The crystal structure of DynF from the dynemicin-biosynthesis pathway of *Micromonospora chersina*

**Abigael J. Kosgei, Mitchell D. Miller, Minakshi Bhardwaj, Weijun Xu, Jon S. Thorson, Steven G. Van Lanen and George N. Phillips**

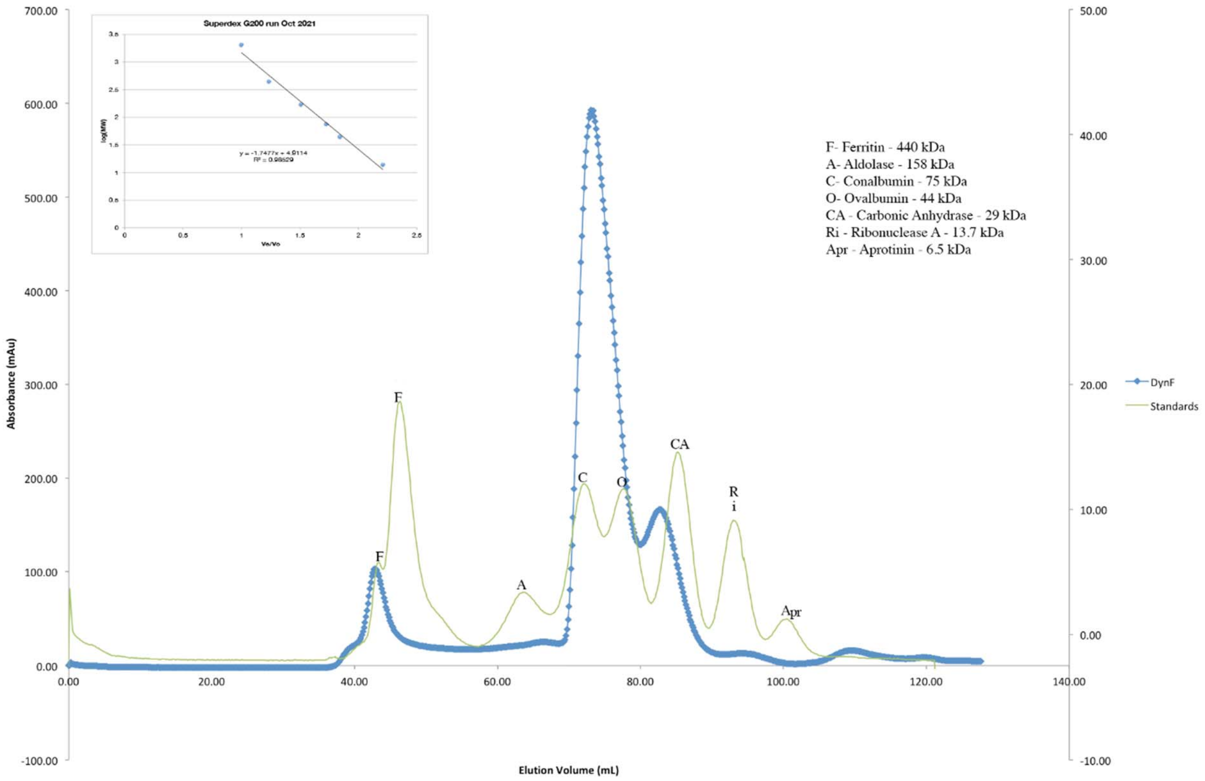


**Figure S1** Elution profiles of DynF with the standards from a Superdex 200 pg gel filtration column. DynF eluted with two peaks from the SEC column. Gel filtration calibration kits (HMW, LMW) were used as standards to determine the oligomeric state of DynF. Based on the sequence, DynF is 22.6 kDa in size. The major elution peak is at the conalbumin (75 kDa) and ovalbumin (44kDa) elution position while the minor peak elutes close to the carbonic anhydrase peak (29 kDa). DynF elutes at a higher oligomeric state.

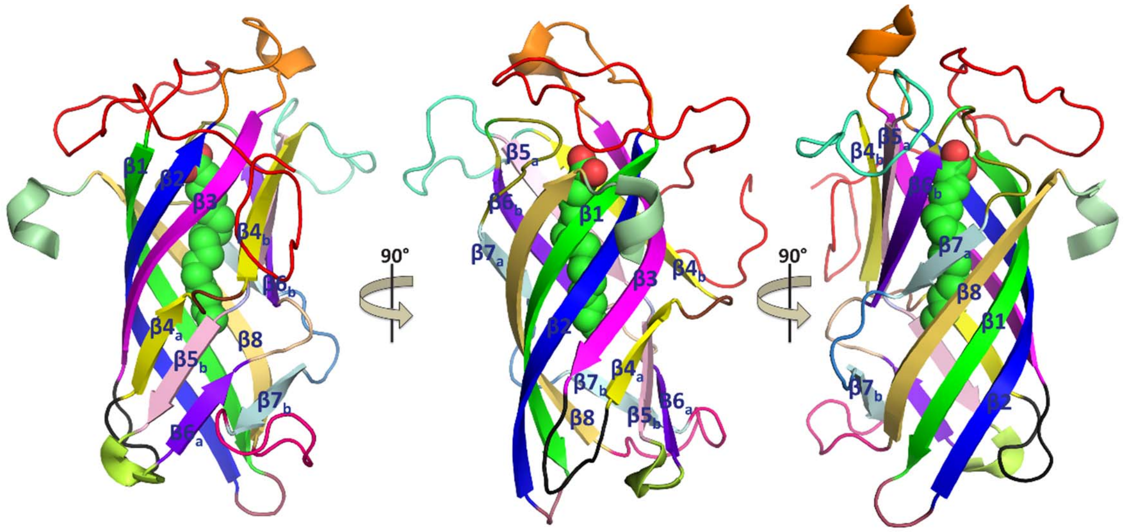


**Figure S2** DynF is an eight-stranded beta-barrel structure with a ligand embedded in the cavity. Three views of the protomer rotated 90° between each view. The beta strands are color-coded and labeled to aid in tracing each of the strands surrounding the ligand.

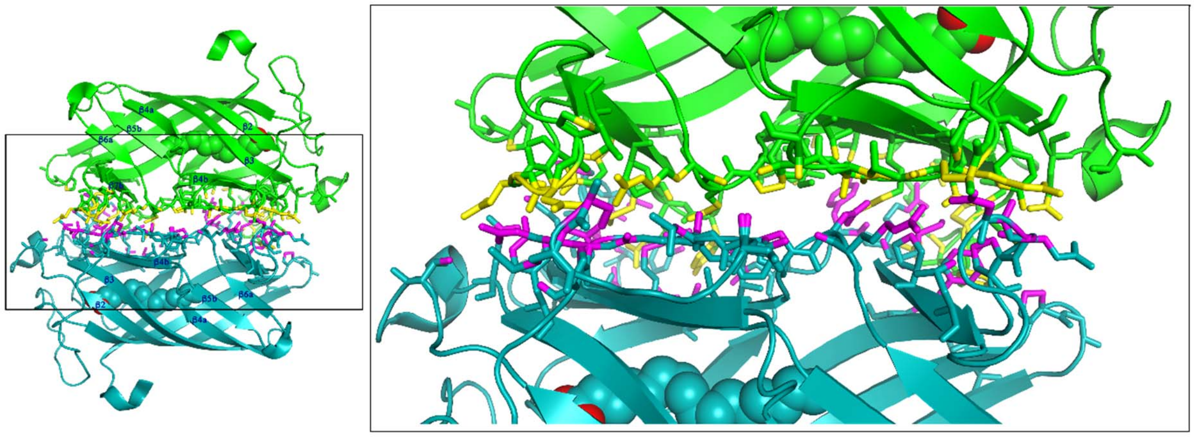

**Figure S3** PyMOL representation of the DynF dimer interface. The left figure shows a ribbon diagram of the DynF dimer. The residues located within 3.5Å of the neighboring protomer at the dimer interface are represented as sticks and colored in yellow and magenta. The right figure represents a zoomed in detail of the dimerization interface. Some Loop residues from beta strands 6 and 7 are also within the dimerization interface.

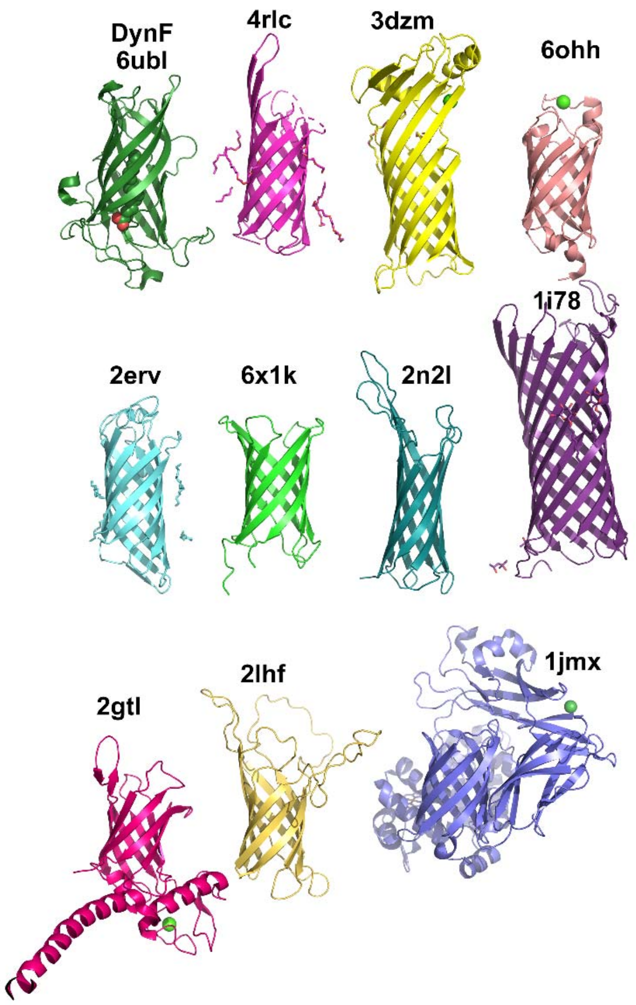


**Figure S4** *DALI* server hits of structures with the highest z-scores from PDB25. Ribbon representation of the top ten hits of structures from PDB25 with highest z-scores (Table S3). The similarity to DynF is based on the presence of beta barrel in their overall structure.

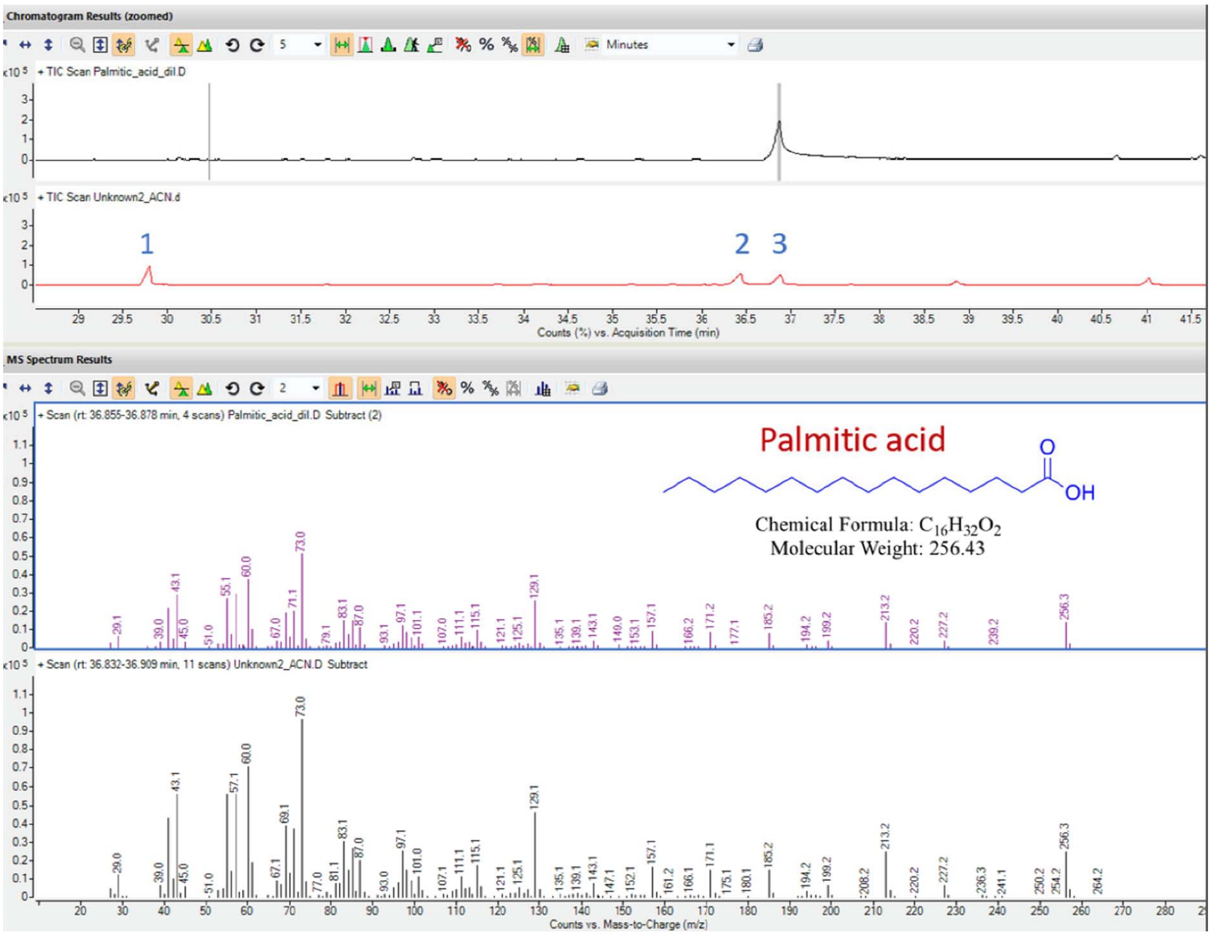


**Figure S5** DynF ligand analysis._Using GC-MS, three peaks were observed in the acetonitrile extract of DynF protein. Peak 3 had the same retention time and identical fragmentation pattern as authentic palmitic acid (Sigma Aldrich - Merck KGaA, Darmstadt, Germany). However, we were not able to identify peak 1 and peak 2.

**Table S1** Residue-residue interactions at the DynF dimer interface I

The table shows hydrogen bonds and salt bridges formed by residues within the dimer interface. Colored in red are the loop residues within β strands 6 (R150, E151, P152) and 7 (M177, R176, Y175, Q174) at the dimer interface.

| Hydrogen bonds | | | | Salt bridges | | | |
|---|---|---|---|---|---|---|---|
| ## | Structure 1 | Dist. [Å] | Structure 2 | ## | Structure 1 | Dist. [Å] | Structure 2 |
| 1 | B:MET 1[ H ] | 2.07 | A:ASP 187[ OD2] | 1 | B:ARG 131[ NH1] | 2.82 | A:GLU 151[ OE1] |
| 2 | B:ARG 10[ HE ] | 2.16 | A:ASP 187[ O ] | 2 | B:ARG 131[ NH1] | 3.71 | A:GLU 151[ OE2] |
| 3 | B:TYR 81[ HH ] | 2.20 | A:GLN 189[ OE1] | 3 | B:ARG 131[ NH2] | 3.45 | A:GLU 151[ OE1] |
| 4 | B:GLN 112[HE22] | 2.22 | A:TYR 193[ OH ] | 4 | B:ARG 131[ NH2] | 2.80 | A:GLU 151[ OE2] |
| 5 | B:TYR 120[ HH ] | 2.03 | A:TYR 175[ OH ] | 5 | B:ARG 176[ NE ] | 3.40 | A:ASP 118[ OD2] |
| 6 | B:ARG 131[HH12] | 1.97 | A:GLU 151[ OE1] | 6 | B:ARG 176[ NE ] | 2.91 | A:ASP 118[ OD1] |
| 7 | B:ARG 131[HH22] | 1.97 | A:GLU 151[ OE2] | 7 | B:ARG 176[ NH1] | 2.88 | A:ASP 158[ OD1] |
| 8 | B:ARG 150[HH11] | 2.24 | A:GLY 9[ O ] | 8 | B:ARG 176[ NH1] | 2.81 | A:GLU 123[ OE2] |
| 9 | B:TYR 175[ HH ] | 1.76 | A:ASP 158[ OD2] | 9 | B:ARG 176[ NH1] | 3.68 | A:GLU 123[ OE1] |
| 10 | B:ARG 176[ HE ] | 2.10 | A:ASP 118[ OD1] | 10 | B:ARG 176[ NH2] | 3.87 | A:ASP 118[ OD2] |
| 11 | B:ARG 176[HH11] | 2.17 | A:ASP 158[ OD1] | 11 | B:ARG 176[ NH2] | 3.62 | A:GLU 123[ OE2] |
| 12 | B:ARG 176[HH12] | 1.96 | A:GLU 123[ OE2] | 12 | B:ARG 176[ NH2] | 3.45 | A:ASP 118[ OD1] |
| 13 | B:ARG 176[HH21] | 2.19 | A:ASP 118[ O ] | 13 | B:ARG 176[ NH2] | 2.95 | A:GLU 123[ OE1] |
| 14 | B:ARG 176[HH22] | 2.09 | A:GLU 123[ OE1] | 14 | B:GLU 151[ OE1] | 3.82 | A:ARG 131[ NE ] |
| 15 | B:ASP 187[ O ] | 2.04 | A:ARG 10[ HE ] | 15 | B:GLU 151[ OE2] | 3.08 | A:ARG 131[ NE ] |
| 16 | B:TYR 193[ OH ] | 2.17 | A:GLN 112[HE22] | 16 | B:GLU 151[ OE1] | 2.78 | A:ARG 131[ NH2] |
| 17 | B:GLU 151[ OE2] | 2.23 | A:ARG 131[ HE ] | 17 | B:GLU 151[ OE2] | 3.48 | A:ARG 131[ NH2] |
| 18 | B:TYR 175[ O ] | 2.18 | A:ARG 131[HH12] | 18 | B:ASP 118[ OD1] | 3.23 | A:ARG 176[ NE ] |
| 19 | B:GLU 151[ OE1] | 1.97 | A:ARG 131[HH21] | 19 | B:ASP 118[ OD2] | 3.34 | A:ARG 176[ NE ] |

| | | | | | | | |
|---|---|---|---|---|---|---|---|
| 20 | B:GLY 9[ O ] | 2.17 | A:ARG 150[HH11] | 20 | B:GLU 123[ OE1] | 3.47 | A:ARG 176[ NH1] |
| 21 | B:ASP 158[ OD2] | 1.74 | A:TYR 175[ HH ] | 21 | B:GLU 123[ OE2] | 2.90 | A:ARG 176[ NH1] |
| 22 | B:ASP 118[ OD1] | 2.49 | A:ARG 176[ HE ] | 22 | B:ASP 158[ OD1] | 2.91 | A:ARG 176[ NH1] |
| 23 | B:GLU 123[ OE2] | 2.04 | A:ARG 176[HH12] | 23 | B:ASP 118[ OD1] | 3.63 | A:ARG 176[ NH2] |
| 24 | B:ASP 158[ OD1] | 2.22 | A:ARG 176[HH11] | 24 | B:GLU 123[ OE1] | 2.75 | A:ARG 176[ NH2] |
| 25 | B:ASP 118[ O ] | 2.08 | A:ARG 176[HH21] | 25 | B:GLU 123[ OE2] | 3.74 | A:ARG 176[ NH2] |
| 26 | B:GLU 123[ OE1] | 1.91 | A:ARG 176[HH22] | 26 | B:ASP 118[ OD2] | 3.83 | A:ARG 176[ NH2] |

**Table S2** Residue-residue interactions at the DynF dimer interface II

The table shows interfacing residues with a buried surface area (BSA, Å$^2$) greater than 0. Values of the accessible surface area (ASA, Å$^2$), solvation energy effect ($\Delta^i$G, kcal/mol) and the buried area percentage (one bar per 10 %) are given as calculated by *PISA*.

<table>
<tr><th>Structure 1</th><th>HSDC</th><th>ASA</th><th>BSA</th><th>Δ<sup>i</sup>G</th><th>Structure 2</th><th>HSDC</th><th>ASA</th><th>BSA</th><th>Δ<sup>i</sup>G</th></tr>
<tr><td>B:SER 0</td><td></td><td>110.76</td><td>22.63 |||</td><td>0.30</td><td>A:SER 0</td><td></td><td>123.96</td><td>16.84 ||</td><td>0.23</td></tr>
<tr><td>B:MET 1</td><td>H</td><td>166.16</td><td>16.72 ||</td><td>0.22</td><td>A:MET 1</td><td></td><td>165.13</td><td>7.99 |</td><td>0.09</td></tr>
<tr><td>B:SER 5</td><td></td><td>93.47</td><td>23.58 |||</td><td>-0.14</td><td>A:SER 5</td><td></td><td>90.26</td><td>14.14 ||</td><td>-0.13</td></tr>
<tr><td>B:VAL 6</td><td></td><td>62.02</td><td>35.96 ||||||</td><td>0.58</td><td>A:VAL 6</td><td></td><td>73.63</td><td>47.53 |||||||</td><td>0.76</td></tr>
<tr><td>B:LEU 7</td><td></td><td>69.87</td><td>43.49 |||||||</td><td>0.09</td><td>A:LEU 7</td><td></td><td>68.67</td><td>45.56 |||||||</td><td>0.17</td></tr>
<tr><td>B:PHE 8</td><td></td><td>42.00</td><td>37.04 |||||||||</td><td>0.27</td><td>A:PHE 8</td><td></td><td>40.49</td><td>35.93 |||||||||</td><td>0.22</td></tr>
<tr><td>B:GLY 9</td><td>H</td><td>36.96</td><td>22.14 ||||||</td><td>0.13</td><td>A:GLY 9</td><td>H</td><td>36.02</td><td>18.40 ||||||</td><td>0.11</td></tr>
<tr><td>B:ARG 10</td><td>H</td><td>141.46</td><td>71.34 ||||||</td><td>-0.37</td><td>A:ARG 10</td><td>H</td><td>144.15</td><td>68.29 |||||</td><td>-0.30</td></tr>
<tr><td>B:PRO 11</td><td></td><td>34.77</td><td>18.53 ||||||</td><td>0.29</td><td>A:PRO 11</td><td></td><td>42.28</td><td>19.66 |||||</td><td>0.31</td></tr>
<tr><td>B:ALA 48</td><td></td><td>23.27</td><td>21.42 ||||||||||</td><td>0.34</td><td>A:ALA 48</td><td></td><td>21.41</td><td>19.74 ||||||||||</td><td>0.32</td></tr>
<tr><td>B:VAL 49</td><td></td><td>29.25</td><td>0.16 |</td><td>0.00</td><td>A:VAL 49</td><td></td><td>31.16</td><td>0.15 |</td><td>0.00</td></tr>
<tr><td>B:PRO 50</td><td></td><td>81.00</td><td>31.80 ||||</td><td>0.51</td><td>A:PRO 50</td><td></td><td>80.18</td><td>33.32 |||||</td><td>0.53</td></tr>
<tr><td>B:GLU 54</td><td></td><td>106.15</td><td>15.81 ||</td><td>-0.11</td><td>A:GLU 54</td><td></td><td>104.30</td><td>14.30 ||</td><td>-0.22</td></tr>
<tr><td>B:THR 71</td><td></td><td>87.85</td><td>17.83 |||</td><td>0.29</td><td>A:THR 71</td><td></td><td>83.51</td><td>3.17 |</td><td>0.05</td></tr>
<tr><td>B:GLU 79</td><td></td><td>40.54</td><td>11.80 |||</td><td>-0.19</td><td>A:GLU 79</td><td></td><td>40.20</td><td>10.61 |||</td><td>-0.17</td></tr>
<tr><td>B:TYR 81</td><td>H</td><td>42.34</td><td>36.88 |||||||||</td><td>-0.11</td><td>A:TYR 81</td><td></td><td>41.02</td><td>31.69 ||||||||</td><td>-0.15</td></tr>
<tr><td>B:ALA 110</td><td></td><td>3.21</td><td>0.00</td><td>0.00</td><td>A:ALA 110</td><td></td><td>2.21</td><td>0.12 |</td><td>-0.00</td></tr>
<tr><td>B:GLN 112</td><td>H</td><td>51.40</td><td>42.06 |||||||||</td><td>-0.50</td><td>A:GLN 112</td><td>H</td><td>54.23</td><td>44.21 |||||||||</td><td>-0.53</td></tr>
<tr><td>B:ARG 114</td><td></td><td>75.62</td><td>47.35 |||||||</td><td>-0.23</td><td>A:ARG 114</td><td></td><td>72.48</td><td>47.19 |||||||</td><td>-0.34</td></tr>
<tr><td>B:THR 115</td><td></td><td>41.08</td><td>0.12 |</td><td>-0.00</td><td>A:THR 115</td><td></td><td>52.96</td><td>0.12 |</td><td>-0.00</td></tr>
</table>

| | | | | | | | | | |
|---|---|---|---|---|---|---|---|---|---|
| B:PRO 116 | | 21.71 | 15.81 | 0.25 | A:PRO 116 | | 19.91 | 14.23 | 0.23 |
| B:PHE 117 | | 173.95 | 119.90 | 1.86 | A:PHE 117 | | 169.91 | 122.49 | 1.78 |
| B:ASP 118 | HS | 95.14 | 31.90 | -0.25 | A:ASP 118 | HS | 93.69 | 36.04 | -0.31 |
| B:TYR 120 | H | 127.94 | 33.83 | -0.21 | A:TYR 120 | | 184.96 | 0.00 | 0.00 |
| B:ALA 121 | | 113.52 | 0.00 | 0.00 | A:ALA 121 | | 74.27 | 5.02 | 0.08 |
| B:GLU 123 | HS | 8.35 | 2.49 | 0.17 | A:GLU 123 | HS | 35.14 | 5.10 | -0.01 |
| B:ALA 129 | | 27.23 | 18.88 | 0.29 | A:ALA 129 | | 26.55 | 19.52 | 0.29 |
| B:ARG 131 | HS | 106.39 | 89.79 | -0.93 | A:ARG 131 | HS | 115.21 | 101.20 | -1.19 |
| B:MET 132 | | 20.49 | 0.00 | 0.00 | A:MET 132 | | 23.22 | 0.15 | 0.00 |
| B:ALA 133 | | 5.35 | 4.68 | 0.07 | A:ALA 133 | | 10.18 | 9.87 | 0.16 |
| B:ARG 150 | H | 127.15 | 97.83 | -0.28 | A:ARG 150 | H | 128.46 | 103.56 | -0.62 |
| B:GLU 151 | HS | 89.12 | 78.54 | 0.07 | A:GLU 151 | HS | 88.33 | 77.06 | 0.00 |
| B:PRO 152 | | 39.54 | 33.31 | 0.53 | A:PRO 152 | | 36.26 | 32.97 | 0.53 |
| B:ILE 154 | | 38.74 | 16.57 | 0.27 | A:ILE 154 | | 42.10 | 17.07 | 0.27 |
| B:THR 156 | | 21.27 | 14.84 | 0.23 | A:THR 156 | | 16.17 | 12.53 | 0.20 |
| B:ASP 158 | HS | 66.05 | 23.85 | -0.33 | A:ASP 158 | HS | 81.22 | 37.38 | -0.35 |
| B:LYS 171 | | 95.01 | 0.00 | 0.00 | A:LYS 171 | | 89.00 | 4.36 | 0.01 |
| B:GLN 173 | | 100.28 | 41.63 | -0.03 | A:GLN 173 | | 91.17 | 40.49 | -0.00 |
| B:ILE 174 | | 26.48 | 0.50 | 0.01 | A:ILE 174 | | 28.52 | 0.00 | 0.00 |
| B:TYR 175 | H | 187.45 | 118.10 | 0.88 | A:TYR 175 | H | 187.91 | 125.19 | 1.02 |
| B:ARG 176 | HS | 186.91 | 143.89 | -1.29 | A:ARG 176 | HS | 176.69 | 158.17 | -3.93 |
| B:MET 177 | | 106.53 | 103.36 | 2.06 | A:MET 177 | | 111.33 | 107.93 | 2.16 |
| B:MET 178 | | 7.37 | 0.67 | 0.01 | A:MET 178 | | 5.20 | 0.00 | 0.00 |
| B:PRO 179 | | 31.10 | 16.74 | 0.27 | A:PRO 179 | | 39.97 | 20.59 | 0.33 |

| | | | | | | | | | |
|---|---|---|---|---|---|---|---|---|---|
| B:LEU 181 | | 12.05 | 1.67 ǀǀ | 0.03 | A:LEU 181 | | 14.06 | 6.03 ǀǀǀǀǀ | 0.10 |
| B:PRO 186 | | 47.92 | 0.37 ǀ | -0.00 | A:PRO 186 | | 43.74 | 2.94 ǀ | 0.02 |
| B:ASP 187 | H | 120.52 | 77.59 ǀǀǀǀǀǀǀ | -0.49 | A:ASP 187 | H | 122.18 | 95.42 ǀǀǀǀǀǀǀǀ | -0.50 |
| B:GLY 188 | | 24.32 | 14.55 ǀǀǀǀǀǀ | 0.05 | A:GLY 188 | | 25.76 | 19.39 ǀǀǀǀǀǀǀǀ | 0.06 |
| B:GLN 189 | | 156.25 | 45.30 ǀǀǀ | 0.08 | A:GLN 189 | H | 162.23 | 85.18 ǀǀǀǀǀǀ | -0.27 |
| B:PRO 190 | | 37.14 | 23.60 ǀǀǀǀǀǀǀ | 0.38 | A:PRO 190 | | 39.75 | 23.12 ǀǀǀǀǀǀ | 0.37 |
| B:TYR 193 | H | 45.19 | 42.96 ǀǀǀǀǀǀǀǀǀǀ | 0.02 | A:TYR 193 | H | 46.05 | 43.60 ǀǀǀǀǀǀǀǀǀǀ | 0.06 |
| B:THR 195 | | 29.89 | 9.37 ǀǀǀǀ | 0.15 | A:THR 195 | | 31.97 | 9.62 ǀǀǀǀ | 0.15 |

**Table S3** Comparison of solved structures in PDB25 to that of DynF in *DALI* server

| Chain | Dali-z | rmsd | lali | nres | % id | Description |
|---|---|---|---|---|---|---|
| 4rlc-A | 9.4 | 2.4 | 115 | 135 | 7 | OUTER MEMBRANE PORIN F |
| 3dzm-A | 9.1 | 2.6 | 116 | 208 | 4 | HYPOTHETICAL CONSERVED PROTEIN |
| 6ohh-B | 9.0 | 2.9 | 115 | 128 | 5 | EF1P2_MFAP2B |
| 2erv-A | 8.9 | 3.2 | 124 | 150 | 5 | HYPOTHETICAL PROTEIN PAER03002360 |
| 1jmx-A | 8.3 | 2.9 | 107 | 493 | 8 | AMINE DEHYDROGENASE |
| 6x1k-A | 7.7 | 3.1 | 109 | 124 | 7 | DE NOVO DESIGNED TRANSMEMBRANE BETA-BARREL TMB2.3 |
| 2n2l-A | 7.4 | 3.3 | 125 | 156 | 4 | OUTER MEMBRANE PROTEIN |
| 1i78-A | 6.9 | 3.1 | 134 | 297 | 7 | PROTEASE VII |
| 2gtl-N | 6.8 | 3.5 | 113 | 220 | 5 | EXTRACELLULAR GLOBIN 4 |
| 2lhf-A | 6.7 | 2.8 | 109 | 178 | 5 | OUTER MEMBRANE PROTEIN H1 |
